# Supplementary material for: Carrying Temoporfin with Human Serum Albumin: A New Perspective for Photodynamic Application in Head and Neck Cancer
Source: Biomolecules. 2022 Dec 29;13(1):68. doi: 10.3390/biom13010068 (PMC9855801; doi:10.3390/biom13010068)
Supplement: Supplementary file 1 [file biomolecules-13-00068-s001.zip › biomolecules-2030899-supplementary.pdf]

Carrying Temoporfin with Human Serum Albumin: A New Perspective for Photodynamic Application in Head and Neck Cancer

Edoardo Jun Mattioli[1 †], Luca Ulfo[2 †], Alessia Marconi[1], Valentina Pellicioni[3], Paolo Costantini[2], Tainah Dorina Marforio[1], Matteo Di Giosia[1], Alberto Danielli[2], Carmela Fimognari[3], Eleonora Turrini[3*] and Matteo Calvaresi[1*]

[1] Dipartimento di Chimica "Giacomo Ciamician", Alma Mater Studiorum—Università di Bologna, Via Francesco Selmi 2, 40126 Bologna, Italy

[2] Dipartimento di Farmacia e Biotecnologie, Alma Mater Studiorum—Università di Bologna, Via Francesco Selmi 3, 40126 Bologna, Italy

[3] Dipartimento di Scienze per la Qualità della Vita, Alma Mater Studiorum—Università di Bologna, Corso d'Augusto 237, 47921 Rimini, Italy

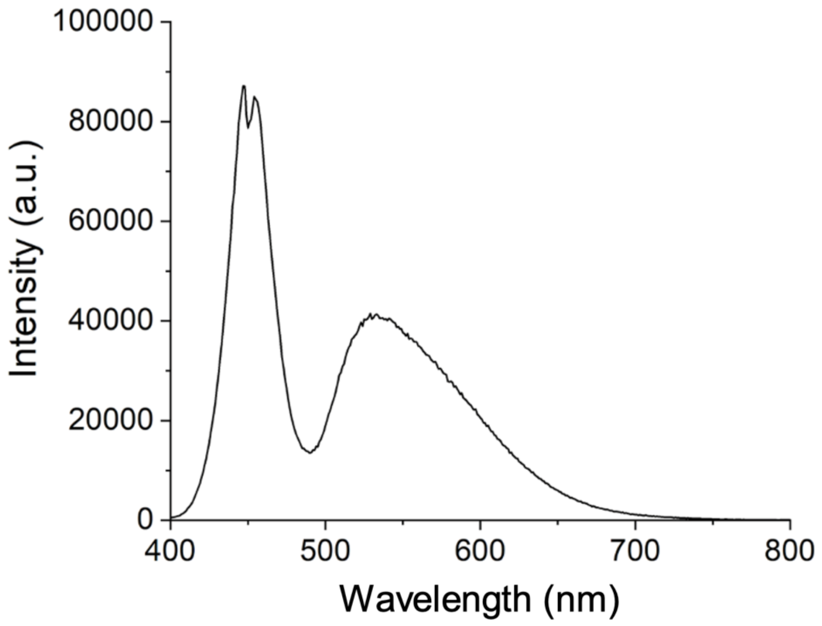

Figure S1. Spectral profiles of the visible light source (Valex 30W, 6500 K, cold white LED) obtained from Edinburgh Analytical Instruments FLS920 spectrofluorimeter.

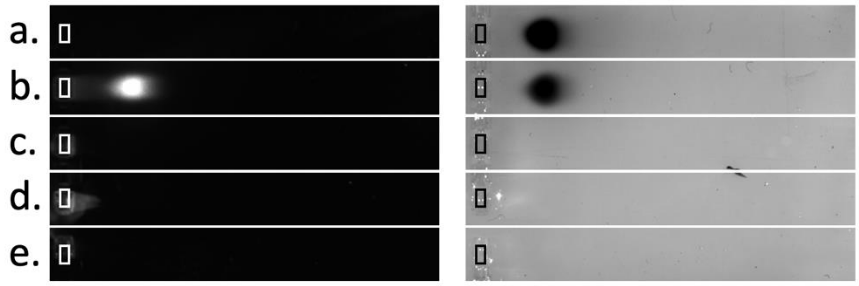

Figure S2. Agarose gel electrophoresis of a) HSA, b) HSA@mTHPC, c) mTHPC from DMSO in PBS, d) mTHPC in 100% EtOH/PG and e) mTHPC from EtOH/PG in PBS. The gel images were acquired before staining, in fluorescent mode (left) and after staining with Coomassie Blue, in colorimetric mode (right).

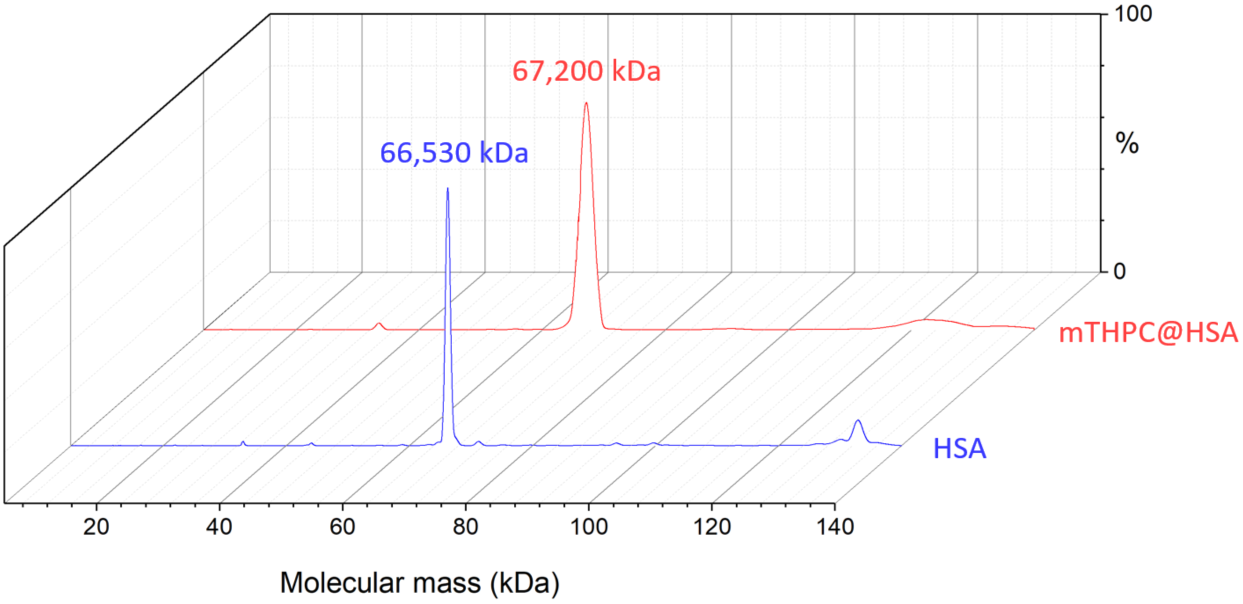

Figure S3. Deconvolution of native mass spectra of HSA before (blu) and after (red) the complexation with mTHPC obtained by the ESI-QTOF-MS

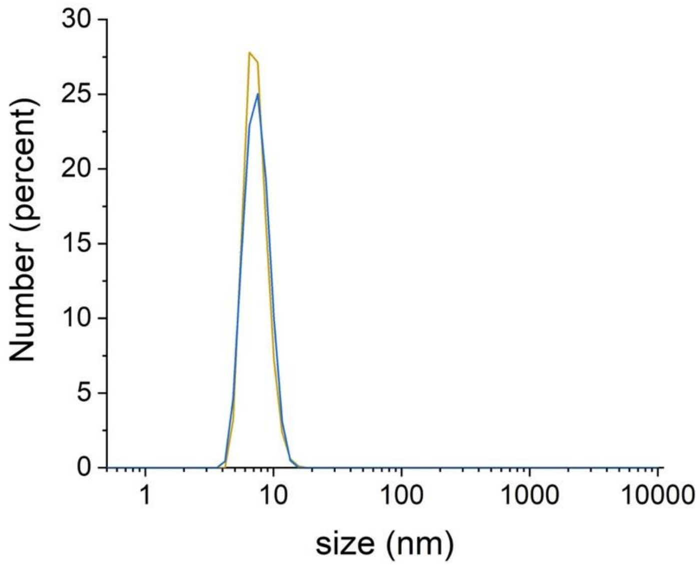

Figure S4. Particle number size distribution of HSA (gold line) and mTHPC@HSA (blue line) in PBS.

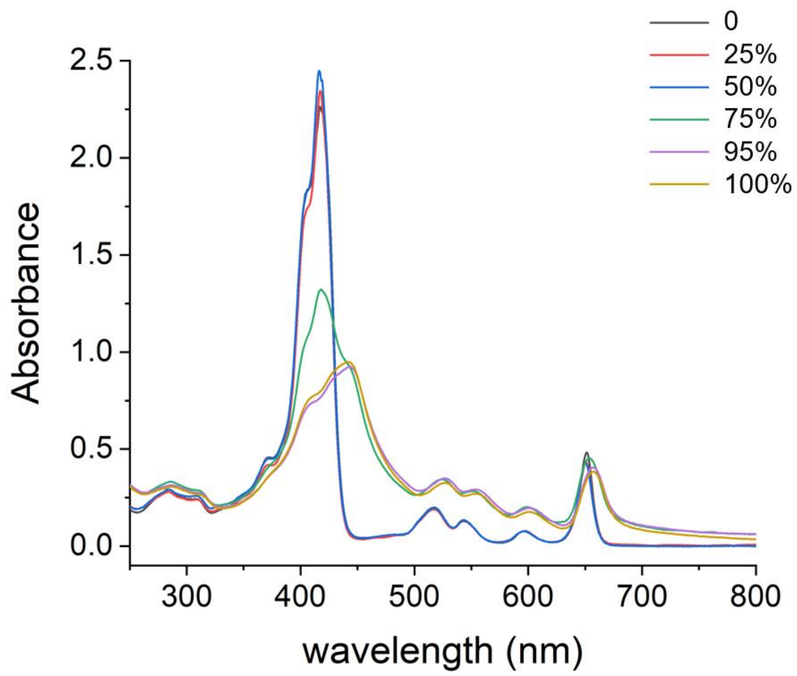

Figure S5. UV-Vis spectra of equimolar amount of mTHPC in the clinical formulation, i.e ethanol/propylene glycol (40/60, w/w) (black line), or with 25% (red line), 50% (blue line), 75% (green line), 95% (violet line) of PBS or in pure PBS (gold line).

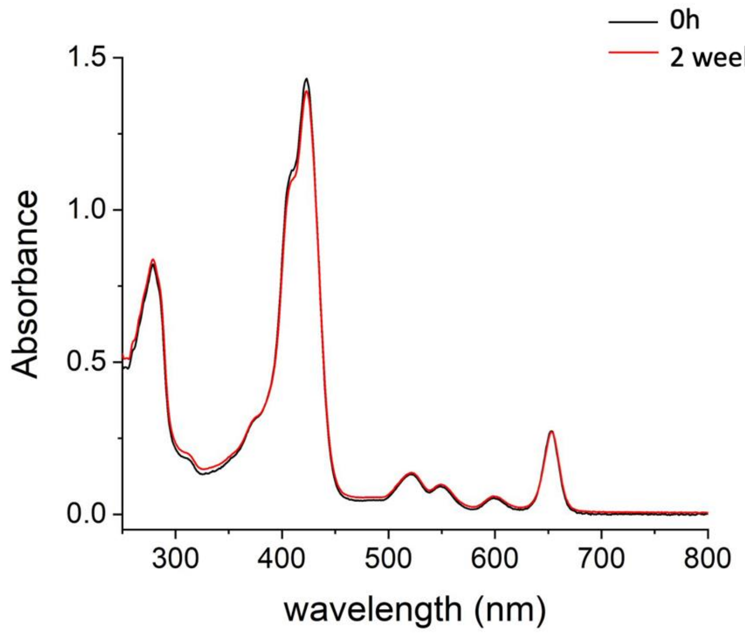

Figure S6. UV-Vis spectra of mTHPC@HSA freshly prepared (black line) and after 2 weeks of storage at 4°C in dark conditions (red line).

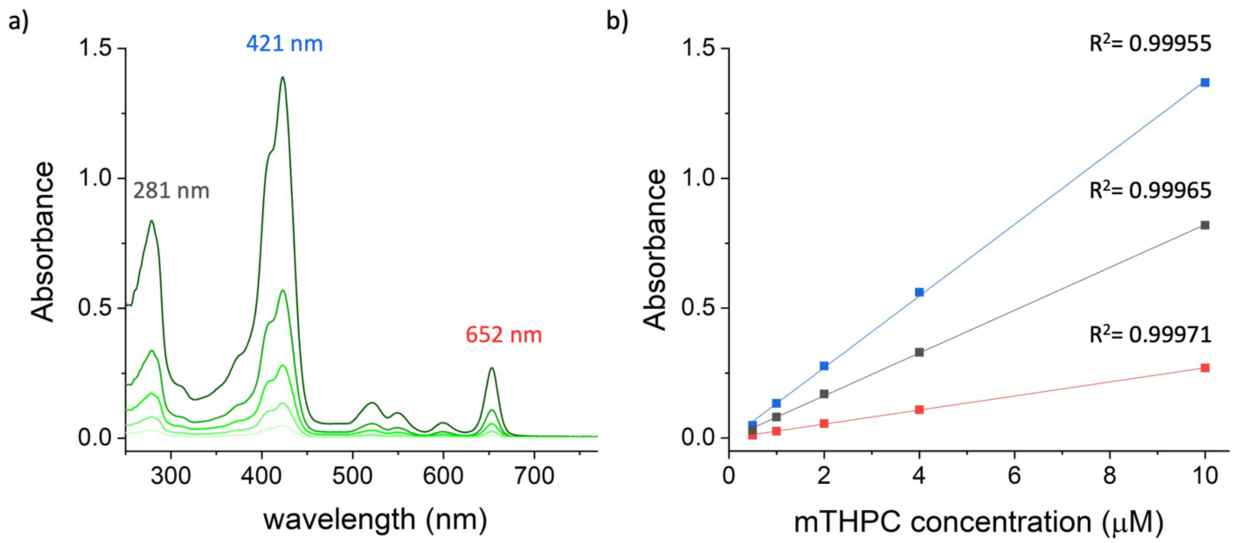

Figure S7. a) UV-Vis spectra of mTHPC@HSA and b) linear relationship after serial dilution from the stock solution (10 µM, 5 µM, 2 µM, 1 µM, 0,5 µM)

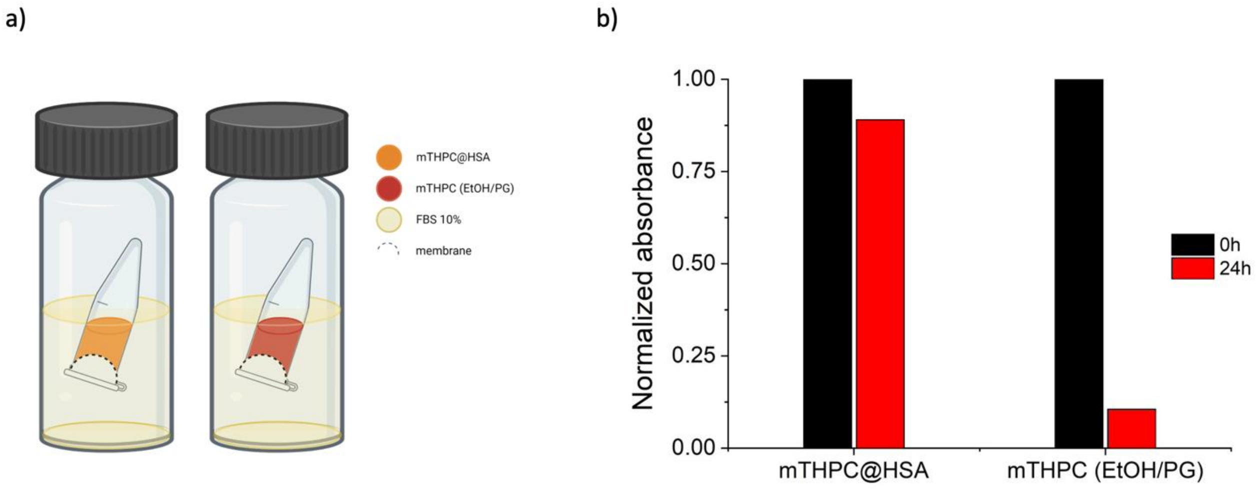

Figure S8. a) Schematic representation of the equilibrium dialysis experiment, performed following an incubation time of 24 h. b) Quantification of the mTHPC retained in the Eppendorf after the equilibrium dialysis experiment based on UV-Vis measurements.

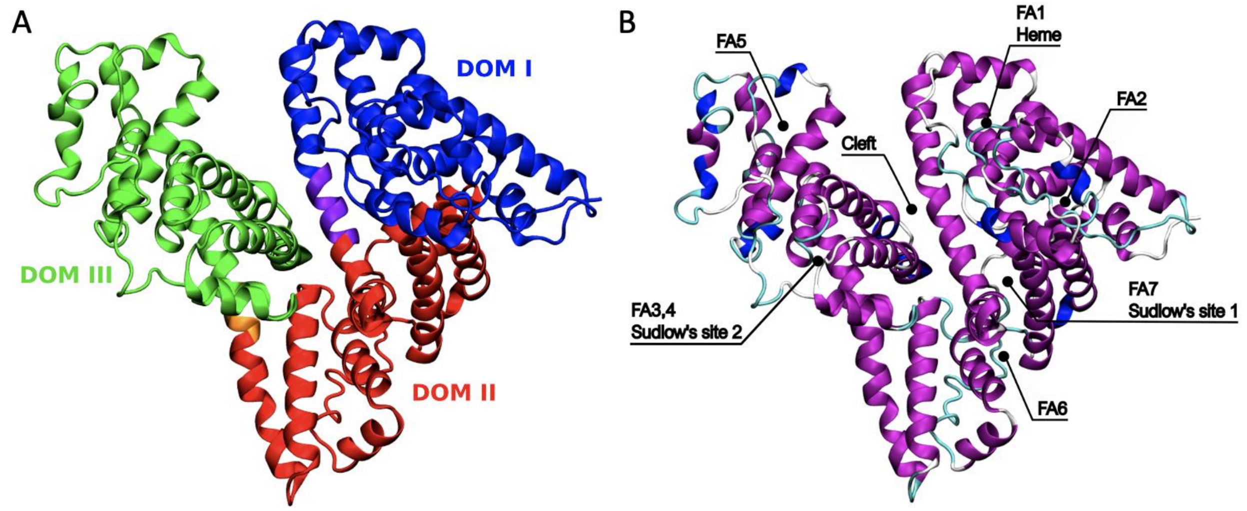

Figure S9. A) Structure of human serum albumin consisting of three domains: Domain I (in blue), Domain II (in red) Domain III (in green). B) Representation of the binding sites identified in human serum albumin.

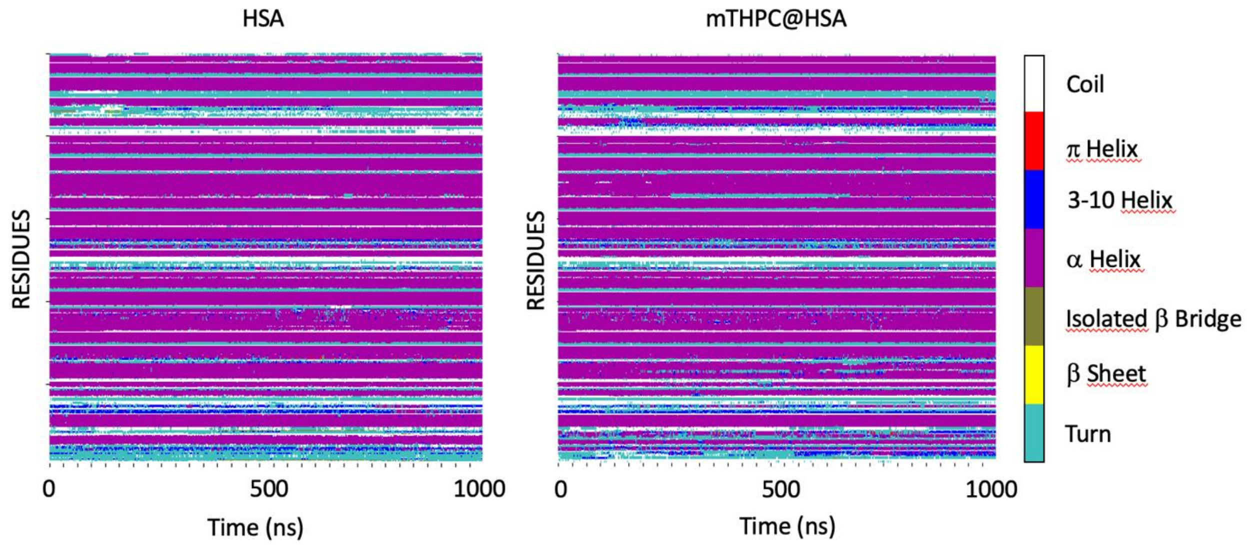

Figure S10. Secondary structure analysis of HSA (on the left) and mTHPC@HSA (on the right) by using VMD timeline for 1 μs simulations.
